# Simultaneous phylogeny reconstruction and multiple sequence alignment

**DOI:** 10.1186/1471-2105-10-S1-S11

**Published:** 2009-01-30

**Authors:** Feng Yue, Jian Shi, Jijun Tang

**Affiliations:** 1 Ludwig Institute for Cancer Research, UCSD School of Medicine, 9500 Gilman Drive, La Jolla, CA 92093, USA; 2 Department of Computer Science and Engineering, University of South Carolina, Columbia, SC 29208, USA

## Abstract

**Background:**

A phylogeny is the evolutionary history of a group of organisms. To date, sequence data is still the most used data type for phylogenetic reconstruction. Before any sequences can be used for phylogeny reconstruction, they must be aligned, and the quality of the multiple sequence alignment has been shown to affect the quality of the inferred phylogeny. At the same time, all the current multiple sequence alignment programs use a guide tree to produce the alignment and experiments showed that good guide trees can significantly improve the multiple alignment quality.

**Results:**

We devise a new algorithm to simultaneously align multiple sequences and search for the phylogenetic tree that leads to the best alignment. We also implemented the algorithm as a C program package, which can handle both DNA and protein data and can take simple cost model as well as complex substitution matrices, such as PAM250 or BLOSUM62. The performance of the new method are compared with those from other popular multiple sequence alignment tools, including the widely used programs such as ClustalW and T-Coffee. Experimental results suggest that this method has good performance in terms of both phylogeny accuracy and alignment quality.

**Conclusion:**

We present an algorithm to align multiple sequences and reconstruct the phylogenies that minimize the alignment score, which is based on an efficient algorithm to solve the median problems for three sequences. Our extensive experiments suggest that this method is very promising and can produce high quality phylogenies and alignments.

## Background

Multiple sequence alignment is one of the most fundamental and important issues in computational biology, and its applications include homologous genes identification, protein structure prediction and phylogenetic reconstruction. The most popular and commonly used approach for multiple sequence alignment is progressive alignment. Basically, it works by aligning the two closest sequences first and adding the remaining sequences one by one until all sequences have been aligned. ClustalW [1,2] is one of the best-known sequence alignment tools based on progressive approach. The main problem with ClustalW is that the initial pairwise alignments are fixed, and early errors cannot be corrected later, even if those alignments conflict with sequences added later [3]. T-Coffee is another popular sequence alignment tool and can be viewed as a variant of the progressive method. It has been reported to get the highest scores on BAliBASE benchmark database [4]. The significant improvement is achieved by pre-processing a data set of all pair-wise alignments and thus allowing for much better use of information in early stages. Roshan et al. [5] later showed that the quality of progressive alignment can be improved by using high-quality guide trees.

On the other hand, phylogeny is the evolutionary history among organisms. To date, sequence data is still the most used data type for phylogenetic reconstruction, and Maximum Parsimony (MP) and Maximum Likelihood (ML) are commonly used as the optimization criteria for reconstructing phylogenies. The most common approach for phylogenetic analysis is usually a two-step process: first, the input DNA or protein sequences are aligned with a multiple sequence alignment program, such as ClustalW and T-Coffee; then, the phylogeny will be inferred from the alignment using tools such as PAUP and RAxML. Generally speaking, most phylogenetic reconstruction methods assume a fixed alignment of the input sequences, which is known to have impact on the accuracy of the inferred phylogeny [6,7]. A set of new methods using direct optimization approach have attracted much attention in the past several years, because such approach requires no prior knowledge of multiple sequence alignment. POY [8] is one of the best known direct optimization methods. However simulations showed that it is inferior to the traditional approach of using MP or ML on aligned sequences [9], as well as on the accuracy of inferred phylogenies [10]. In this paper, we will present a new direct optimization method that is based on affine gap models and uses an iterative approach solving many instances of median problems of three sequences. Our simulations show that this method is superior to the traditional approach of phylogenetic reconstruction based on prior alignment; while for sequences with high substitution rates, it is also able to produce better multiple sequence alignment than those widely used sequence alignment tools.

### Multiple sequence alignment

An important way to compare multiple sequences is *tree alignment*, which is motivated by the fact that in most cases the sequences are not independent of each other but rather related by a evolutionary tree [11]. The tree alignment problem was developed principally by Sankoff, who also proposed the first exact (exponential-time) algorithm [12] via dynamic programming.

However, finding sequence assignment of the internal nodes that maximizes the similarity score is NP-hard [13]. Various approximation algorithms have also been designed to heuristically compute tree alignments and phylogenies, such as TAAR [14] and GESTALT [15]. All of these heuristics compute the alignment along a given tree [16] or a simple tree such as the neighbor-joining [17] tree and minimum spanning tree [15].

Tree alignment can be further improved with the iterative method proposed by [18]. For any binary tree, each internal node has three neighbors. Re-optimization for the internal nodes can be achieved by iteratively relabeling each of them using the three neighboring nodes. The process will stop when no further improvement is possible. In our experiment, the tree will always converge after only several iterations. Therefore, how to compute a high quality alignment for three sequences and infer their internal sequence is essential.

### Median problem of three sequences

For *n *sequences {*S*_1_, *S*_2_,...,*S*_*n*_}, the *median problem *is to find a sequence *S*_0 _such that $\sum_{i=1}^{n}d_{0i}$ is minimized, where *d*_0*i *_is the distance between *S*_0 _and *S*_*i*_. When *n *= 3 we will call this the *median problem of three*, or just the *median problem*. The median problem is of particular importance since the smallest binary tree has only three leaves. Gotoh [19] presented the first three sequences alignment algorithm under affine gap model. Powell et al. [20] presented an algorithm to infer optimal alignments based on tree score by employing Finite State Machines (FSM), which are explicitly used for the generation of the three sequences from a parent sequence. However, the running time and memory space usage in both algorithms are *O*(*n*^3^), where *n *is the length of the sequence. The limitation is obvious – huge demand of memory space makes it impossible to work for sequences with length of more than a couple of hundred characters. For example, when *n *= 300, the total memory usage will be around 3 G bytes, and when *n *= 1000, the total memory usage will be over 100 G bytes, which are way over current workstation's capacity.

Powell et al. [20] presented another algorithm to tackle the memory usage problem. The memory complexity of the new version is *O*(*d*^3^), where d is the tree score of the alignment. It is highly efficient when *d *<<*n*, which requires the input sequences be very similar, and the cost model be simple. Thus their algorithm cannot use complex substitution matrix such as PAM [21] or BLOSUM [22], where the cost of substitutions can be very high, resulting in very large distances *d *that can easily grow much larger than *n*. Later, Yue and Tang [23] proposed an algorithm that solved the high memory usage by applying a divide-and-conquer strategy. This median solver reduces the memory usage to *O*(*n*^2^) while still producing the optimal alignment, which will be used as the core of our method presented in this paper.

## Methods

### Algorithm overview

Our new algorithm takes *k *un-aligned sequences as input and conducts a search to find the best tree with lowest score. It then reports this tree as the phylogeny. As a by-product of this procedure, we will also produce a multiple alignment with respect to the best tree. There are two major components in this algorithm: 1) a procedure to score a given tree and produce alignment; and 2) a strategy to find the best tree from all possible trees. For a tree with *k *leaf nodes, we can assign each leaf (external) node with one of the given sequences. When the sequences of all *k *- 2 internal nodes are also known, we can easily obtain the *tree score *by summing all edge lengths induced by the pairwise alignment score between the two sequences at both ends of the edge. However, since we do not know any information about the internal sequences, we must explicitly *label *the internal nodes with sequences that produce the minimum tree score, which is computationally very hard. One should note that unlike some other alignment packages, we assume matches to have zero cost, and mismatch and gaps penalties to be positive, thus the best alignment will have the smallest score.

### Scoring a given tree

For a given tree, each input sequence is assigned to a leaf node with respect to the tree topology. Our strategy for tree labeling and scoring consists of two main steps: 1) initialize each internal node with some sequence; 2) iteratively refine the internal sequences until no further change occurs.

We need to assign each internal node an initial sequence to start our scoring procedure. This can be done by simply assigning each internal node a random string of DNA or protein sequences. However, other complex procedures will yield much better results. In our algorithm, a better initialization method is devised by assigning each internal node as the median solution from its three nearest leaves (in term of topology), using the median solvers discussed in the next section. We will arbitrarily pick one set of leaves if there are multiple choices of nearest neighboring leaves.

#### Solving the median problem

The inputs to the median problem are three sequences, *A*, *B *and *C *of length *X*, *Y *and *Z *respectively. The output is three aligned sequences, *A'*, *B' *and *C' *of the same length *L*, as well as an aligned median sequence *M' *(with length *L*). The median sequence *M *can then be obtained by simply removing all gaps from *M'*.

There is a straight-forward solution for the median problem using dynamic programming technique [20]. Assume each of the three sequences is generated independently from their common parent sequence (the median) by a three-state Finite State Machine (FSM), and the possible states for the FSMs are I (insert), D (delete) and M (match/mismatch). The problem of aligning sequences is then transformed into finding how the aligned sequences are generated. At each site in the aligned sequences, there are 27 (3^3^) possible combinations of states (MMM, IMD, ...). We can construct a cube of size *X *× *Y *× *Z *for each combination of states, and the result can be computed directly and is optimal. However, the time and the memory complexities of this simple algorithm are both O(*n*^3^), where *n *is the length of the input sequences. Thus, it is restricted by the high demand of memory usage and can only work on sequences of less than a few hundred characters.

**Figure 1:**
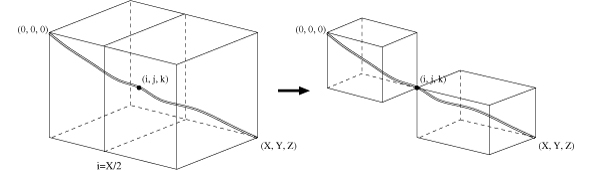
How to split a 3-D cube.

Myers et al. [24] presented a linear space algorithm for pairwise alignment using affine gap costs. Our algorithm uses a similar divide-and-conquer approach to split the three-dimensional cube. Let $i=\frac{X}{2}$, the plane defined by *i *will cut the cube into two halves (Figure 1 left), and we need to find the *midpoint *on plane *i *where the final alignment passes through. Once the midpoint is identified, we will apply the above procedure to the two small cubes, one defined by points (0, 0, 0) and (*i*, *j*, *k*), and the other by (*i*, *j*, *k*) and (*X*, *Y*, *Z*) (Figure 1 right). The process will be executed recursively until boundary conditions are encountered [23].

The exact median solver can produce optimal alignment using *O*(*n*^2^) memory space, which makes it possible to align sequences with several thousand characters. However, the computation time remains *O*(*n*^3^) and it may take several hours to produce an exact median for sequences of length around one thousand characters. Thus this exact solver is not satisfactory for large-scale sequence comparison. To handle longer sequences, we also developed a simple and fast heuristic solution as a substitute. It works as follows: pick up and align the closest two sequences of the three input sequences, and then align the third sequence with the pairwise alignment of the two. The median sequence can be inferred by a consensus vote at each site of the alignment of the three sequences. The time and space complexities of this procedure are both *O*(*n*^2^). As shown in the section of experimental results, although the resulted alignment and internal sequence are not as good as those found by the exact median version, it is thousands of times faster and can be used to produce very high quality phylogenies.

#### Iterative refinement

No matter how we obtain the initial internal sequences, they are surely very far away from optimal solutions, thus we must refine these sequences. Inspired by BPAnalysis [25] and GRAPPA [26] for genome rearrangement analysis, we devised an iterative refinement procedure which works as the following:

• For each internal node *S*_*i *_in the tree with a sequence assignment *M*_*i*_, we compute a new sequence ${M^{\prime }}_{i}$ with its three neighboring nodes (internal and external), using the median procedure described above. If there is any gap in the new node, we will remove all gaps so the next median problems still deal with gapless sequences.

• If the sum of the three new edge lengths is improved (i.e., lower value than the previous sum), we replace the previous assignment of *S*_*i *_using the new computed sequence ${M^{\prime }}_{i}$. Otherwise, we keep the previous assignment (*M*_*i*_) on *S*_*i*_.

• Starting from the root node, we can repeatedly relabel each internal nodes using the above two steps until no further improvement is possible.

Our method iterates the internal nodes through a depth-first procedure, although a breadth-first procedure can also be used. If the tree is un-rooted, we can randomly pick an internal node as the root. In our experiments, we found that this procedure is very robust and generally requires only three to four iterations, and the impact of picking different roots is negligible.

#### Output final tree alignment

Now we have a fully labeled tree with each internal and external node assigned a sequence, thus we can compute the final tree score by simply adding all edge scores. Each edge score can be obtained by conducting pairwise alignment on the two sequences at the end of the edge. However, since we also want to obtain an alignment, we need to compute a multiple sequence alignment with respect to the best tree topology, with a basic assumption that the best tree (the phylogeny) will also produce the best alignment.

Our method of producing the final alignment is similar to those progressive alignment methods. Starting from the root node, we first compute the pairwise alignment between the root and its right child node. After this step, there might be some gap symbols ('-') inserted in the alignment. Next, we need to add a new sequence (for example, the left child node of the root node) to this alignment, using the following steps to align a sequence with an alignment:

• First, we define a special character 'X' with a property that there is no charge of penalty of aligning it with either a character or a gap '-'. Then we replace all the '-' symbol in the alignment with the special character 'X'. Thus, the previous alignment will be transformed into two new sequences with only characters (including the special character) and no gaps.

• We then conduct pairwise alignments between the new sequence and each of the two modified sequence. The best pairwise alignment will be kept as a "pivot", and the other alignments will be discarded. This procedure may introduce some new gaps and as a result, the new alignment will be of a different length with the rest of the previously aligned sequences. In the new alignment, there are two kinds of gaps: 'X', carried gaps from previous alignment; '-', gaps inserted at this step.

• We will verify the position of the newly inserted gaps and add gaps into the same position in the rest of the previously aligned sequences. At this point, the sequence has been aligned with the previous alignment and they are of the same length.

Using the same strategy, whenever a new sequence is added, we align it with each one of the previous aligned cluster and keep the best pairwise alignment as the "pivot" to generate a new alignment cluster. The computation will continue until all the nodes in the tree have been covered.

### Searching the large tree space

Finding the best tree from the large tree space is always very difficult. There are a total of (2*n *- 5)!! = (2*n *- 5) × (2*n *- 7) × ⋯ × 3 un-rooted trees for any tree with *n *leaves. This number grows very fast: there are 3 trees for *n *= 4, two million trees for *n *= 10, but 2^66 ^trees for *n *= 20. To remedy this problem, many heuristics have been developed to search this large space.

Of course, the simplest way to search for the best tree is to enumerate and score all trees. Not all trees need to be scored though, since some trees are clearly very bad and can be safely discarded by checking some lower bounds [26]. For example, there are a suite of circular-order lower bounds derived from triangular inequalities. However, these lower bounds are loose here and not too many trees can be pruned, hence the exhaustive approach does not work for datasets with more than 10 sequences.

Many heuristic tree searching approaches are available, including nearest-neighbor interchanges (NNI), subtree pruning and regrafting (SPR), and tree bisection reconnection (TBR). In NNI, one of the internal edges is chosen at random and the four subtrees (by removing the edge and its two nodes) are reconnected randomly. In SPR, a random edge is selected and two subtrees are created, then one of the two subtrees is removed at random and reinserted along a random edge in the other subtree. In TBR, similar to SPR, one edge is removed and the tree is divided into two subtrees, then they are joined by an edge connecting two midpoints of edges of the two subtrees. All these heuristics require a good start tree. In our experiment, we find that tree returned by distance-based neighbor-joining (NJ) method usually do well on the test dataset, thus we use NJ tree as the start point, and then we run TBR method to generate new trees from the Neighbor-joining tree. Whenever a new tree reports a better score, the best tree is updated and the tree is stored. The whole algorithm will stop when no improvement of tree score is achieved.

Many methods have been developed by researchers to handle the large tree space, including branch-and-bound methods, quartet-based methods and disk-covering methods [27]. The tree search method used by our method can be further improved using these more complex methods.

## Results and discussion

We implement the algorithm as a C program called MSAM and test its accuracy through experiments. MSAM can handle both DNA and protein sequences and allows users to specify different mutation cost matrices such as PAM or BLOSUM series, as well as the costs for gap opening and extension. We also develop MSAM-H, a time-efficient version of MSAM, which adopts the fast heuristic median solver instead of the exact solver during the tree refinement phase. Since phylogeny analysis deals with lost historical information, we concentrate our experiments on simulated datasets, where the true evolutionary history and alignments are known.

### Phylogeny accuracy

The Rose (Random Model of Sequence Evolution) [28] software package is a widely used simulator, which implements the HKY85 model of DNA sequence evolution and allows for insertions and deletions. We use the standard measurement of *false positive *and *false negative *[29] to determine the topological accuracy of a method. If the true tree has an edge defining a bipartition with no equivalent in the reconstructed tree, that edge is a *false negative *(*FN*); conversely, if the reconstructed tree has an edge defining a bipartition with no equivalent in the true tree, that edge is a *false positive (FP)*. The false negative rate is the number of false negative edges divided by the number of edges of the true tree. Since we are dealing with binary trees, FP and FN will be equal, hence only false negative rates are reported here.

In this experiment, we first use ClustalW or T-Coffee to align the input sequences and then use PAUP [30] to generate the Maximum Parsimony tree from the alignment. On the other hand, MSAM is tested directly on the un-aligned input sequences. We test ClustalW, T-Coffee and PAUP with their default parameters (most used), and for MSAM and MSAM-H we use the most common parameters, i.e., match costs 0, mis-match costs 1, gap opening penalty is 3, and gap extension penalty is 1. We also test against POY, which is the most used direct optimization methods.

We use birth-death model trees produced by the r8s software package [31], with random deviation factor multiplied on each edge to deviate the model trees from ultrametric. For each model tree we generate DNA sequences by using ROSE with the following parameters:

• the sequences are over the character set of {A, C, G, T};

• the transition/transversion ratio is set to 2, the mutation frequencies are set as [0.25, 0.25, 0.25, 0.25];

• the insertion/deletion length frequencies are set as [.2, .2, .2, .1, .1, .1, .1], which control the probabilities for gaps of lengths from 1 to 7;

• two groups of the insertion/deletion threshold are tested: 0.001 and 0.005;

• three substitution rates are tested: 0.1, 0.2 and 0.3;

• four different expected sequence lengths are tested: 200, 400, 800 and 1000.

**Figure 2:**
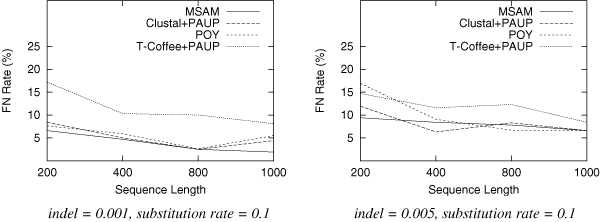
**False negative rate as a function of the sequence length.** Each data set has 10 taxa with substitution rate of 0.1.

**Figure 3:**
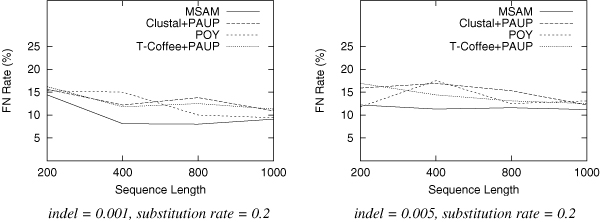
**False negative rate as a function of the sequence length.** Each data set has 10 taxa with substitution rate of 0.2.

**Figure 4:**
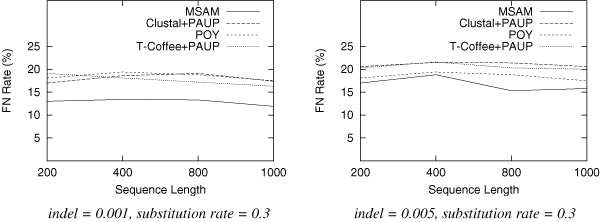
**False negative rate as a function of the sequence length.** Each data set has 10 taxa with substitution rate of 0.3.

We test trees of 10 taxa for each setting of parameters. For each category, we generate 100 dataset and report the average results. Since speed is a major concern here, we use only MSAM-H with the fast heuristic median solver. The results are shown in Figure 2, Figure 3 and Figure 4.

From these figures, we find that for datasets produced in this experiment, MSAM-H outperforms POY, Clustal+PAUP and T-Coffee+PAUP in most of the categories, especially when the substitution rate is increased (sequences are more distant). We also need to point out that the topology accuracy error rate decreases with the increase of input sequence length; the error rate also increases with the increase of indel rate, because for these datasets the alignment is more difficult.

This experiment suggests that even MSAM-H can produce better results than phylogenetic methods using prior knowledge of alignment. The average time for MSAM-H to iteratively score a given tree is less than one second for 200 characters, less than 10 seconds for 800 characters, and less than 25 seconds for 1000 characters. The scoring procedure convergence quickly and all trees require fewer than five iterations to score. On the other hand, the hill-climbing tree search procedure will stop with fewer than 1000 trees being examined. In short, the time used by MSAM-H on 10 taxa ranges from several minutes to an hour. Although more taxa will surely require more time, as we mentioned above, this obstacle can be overcome by using other methods developed in the phylogeny research community.

### Alignment accuracy

We compare the alignments produced by MSAM with those obtained by POY, ClustalW and T-Coffee. We also report the score from MSAM-H. In this experiment, we use the same datasets created in the previous experiments, and the final tree alignments are produced on the best trees found in the previous section. We test ClustalW and T-Coffee with their default parameters and for MSAM and MSAM-H we use the common parameters of match cost 0, mis-match penalty 1, gap opening penalty 3, and gap extension 1. For POY, We used a testing script similar to that suggested by the authors. The alignments are then assessed using bali_score, a program provided by BAliBASE [32] to compare the inferred and the supposedly correct alignments. bali_score reports two scores: SP and CS. SP (Sum-of-Pair) score represents percent of residue pairs correctly aligned, and CS (column score) represents percent of columns correctly aligned. Higher SP and CS scores suggest better performance. In our experiments, although SP and CS scores are different, they will not affect the comparison results of all these programs, hence only SP scores are reported.

Table 1, Table 2 and Table 3 show the average SP scores from these four programs. When the substitute rate is 0.3 (sequences are distant), MSAM almost always achieves the highest score. In fact, even the fast and heuristic version of our program MSAM-H outperforms POY, T-Coffee and ClustalW in this category. However, when the sequences are closer, POY and the traditional methods outperforms MSAM except when the sequences are short, and indeed POY is arguably more accurate than all methods tested here. Compared to the results presented in [23], where for three sequences, the exact median solver is clearly better than ClustalW and T-Coffee, we believe our alignment method can be further improved and more information from the inferred internal sequences should be used. We also observe that the scores of MSAM are better than those of MSAM-H (around 7% to 16%), although they go through identical procedures except for the assignment of internal nodes, which clearly shows that better medians (internal sequences) can yield better alignments.

**Table 1:** SP scores for ClustalW, T-Coffee, MSAM and MSAM-H on substitution rates of 0.1

	*l *= 200		*l *= 400		*l *= 800		*l *= 1000	
*indel*	0.001	0.005	0.001	0.005	0.001	0.005	0.001	0.005

Clustal	0.785	0.408	0.790	**0.439**	0.801	**0.437**	0.779	**0.356**
T-Coffee	0.534	0.419	0.602	0.429	0.615	0.431	0.577	0.333
POY	**0.838**	**0.442**	**0.817**	0.405	**0.805**	0.430	**0.784**	0.320
MSAM-H	0.652	0.334	0.467	0.291	0.489	0.215	0.476	0.187
MSAM	0.754	0.366	0.560	0.328	0.586	0.236	0.572	0.217

**Table 2:** SP scores for ClustalW, T-Coffee, MSAM and MSAM-H on substitution rates of 0.2

subrate	*l *= 200		*l *= 400		*l *= 800		*l *= 1000	
*indel*	0.001	0.005	0.001	0.005	0.001	0.005	0.001	0.005

Clustal	0.441	0.286	0.332	**0.272**	0.350	0.205	0.372	0.173
T-Coffee	0.305	0.247	0.186	0.220	0.262	0.181	0.289	0.153
POY	0.448	0.277	**0.458**	0.264	**0.388**	**0.245**	**0.445**	**0.212**
MSAM-H	0.456	0.264	0.269	0.222	0.178	0.168	0.274	0.133
MSAM	**0.522**	**0.290**	0.289	0.248	0.192	0.183	0.291	0.146

**Table 3:** SP scores for ClustalW, T-Coffee, MSAM and MSAM-H on substitution rates of 0.3

subrate	*l *= 200		*l *= 400		*l *= 800		*l *= 1000	
*indel*	0.001	0.005	0.001	0.005	0.001	0.005	0.001	0.005

Clustal	0.305	0.212	0.221	0.148	0.219	0.130	0.201	0.137
T-Coffee	0.187	0.176	0.128	0.118	0.132	0.092	0.137	0.110
POY	**0.423**	0.209	0.114	0.062	0.228	0.119	0.145	0.041
MSAM-H	0.315	0.203	0.213	0.149	0.225	0.113	0.178	0.132
MSAM	0.359	**0.219**	**0.234**	**0.154**	**0.240**	**0.132**	**0.211**	**0.141**

In terms of running time, ClustalW is always the fastest, followed by MSAM-H, POY and T-COFFEE. Among all these programs, the execution time of MSAM is the longest, largely due to its exact median computation procedure, which has time complexity of *O*(*n*^3^).

## Conclusion

We have presented an algorithm to align multiple sequences and reconstruct the phylogenies that minimize the alignment score. This method is based on efficient algorithms to solve the median problem of three sequences. For more sequences, our method overcomes various computational problems in tree scoring and tree searching. Our extensive experiments suggest that this method is very promising and can produce high quality phylogenies and alignments.

Further improvements are needed, however. We need to find a better method to produce the final alignment with respect to the best tree, and use more complex methods to search through the tree space. More experiments are necessary as well. For example, we plan to compare MSAM with other phylogeny tools such as Maximum Likelihood methods and Bayesian methods.

## Competing interests

The authors declare that they have no competing interests.

## Authors' contributions

All authors contribute to the development and implementation of the algorithms, and FY and JS are in charge of conducting simulations and analyzing results.
